# Variation of pectineus muscle forming a hiatus

**DOI:** 10.1007/s12565-020-00593-5

**Published:** 2021-01-05

**Authors:** Hankyu Kim, Yong Seok Nam

**Affiliations:** 1 Department of Anatomy, College of Medicine, The Catholic University of Korea, Banpo-Daero 222, Seocho-Gu, Seoul, 06591 Korea; 2 The Catholic Institute for Applied Anatomy, College of Medicine, The Catholic University of Korea, Banpo-Daero 222, Seocho-Gu, Seoul, 06591 Korea

**Keywords:** Adductor longus, Hiatus, Pectineus, Variation, Vascular surgery

## Abstract

Knowledge of the anatomic variations in the pectineus muscle is important for vascular surgeons to minimize complications following surgical approach to the distal part of the deep femoral artery. During routine dissection of the thigh, variations in the bilateral pectineus muscles were identified in an 82-year-old male cadaver. On both sides, the superficial and deep layers of the pectineus were divided at its distal part, forming a triangular-shaped hiatus between them and the femur shaft. Distally, the tendon of the superficial part intermingled with the tendon of the adductor longus. The tendon of the deep part was inserted into the pectineal line. On the right side, the deep femoral artery and its first perforating artery passed through the hiatus. On the left side, the deep femoral artery pierced the hiatus, and then, the first perforating artery was branched from the deep femoral artery. No reported case has described a pectineal hiatus. The variations observed in this study are an ontogenetic vestige of the two different origins of the pectineus. The insertion of the superficial layer into the adductor longus tendon suggests a close relationship between these muscles during prenatal development. Surgeons should be aware of the variation to minimize injury to the pectineus muscle while approaching the deep femoral artery.

## Introduction

The pectineus muscle is flat and quadrangular-shaped. This muscle is attached proximally to the superior ramus of the pubis and distally along the pectineal line from the lesser trochanter to the linea aspera (Standring 2016). It appears to be incompletely separated by two layers, superficial and deep (Paterson 1891). The pectineus is mostly innervated by the femoral nerve, but sometimes innervated by the accessory obturator nerve when this nerve is present. Innervation by the obturator nerve is relatively rare (Anagnostopoulou et al. 2009; Paterson 1891; Woodburne 1960).

The deep femoral artery is a large branch arising from the femoral artery distal to the inguinal ligament. It gives off lateral and medial circumflex branches in the proximal thigh, and perforating and muscular branches more distally. The deep femoral artery passes between the pectineus and the adductor longus, and then descends between the adductor longus and the adductor brevis (Standring 2016). The distal part of the deep femoral artery, located behind the adductor longus, might be chosen as an alternative approach site during secondary vascular surgery, particularly when the standard access route via more proximal vessels is encased in extensive scars or infection (Veith et al. 2016).

Variations of the pectineus, of which muscular fibers were partially fused with the adductor longus, were reported (Bardeen 1906; Kumar 2013), but the pectineus muscle has been generally considered as a muscle with few variations (Paterson 1891). Here, we report a case of bilateral variations in the pectineus muscles, forming a hiatus, and its positional relationship with the deep femoral artery.

## Case report

**Fig. 1:**
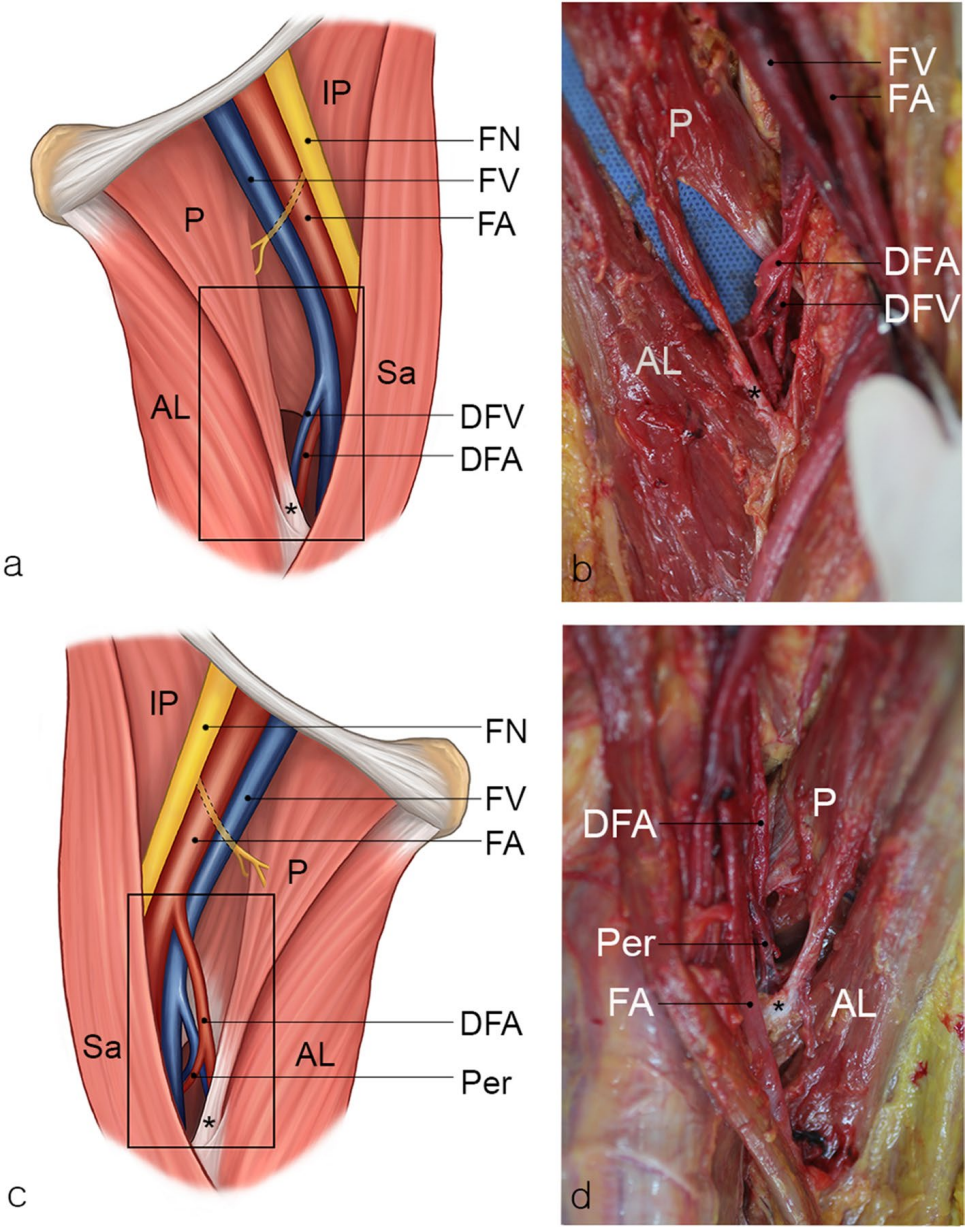
**a**, **b** A case of anatomical variation in the left thigh. A blue cloth was inserted behind the pectineus for clear discrimination. Deep femoral vessels pass through the hiatus formed by the pectineus. **c**, **d** A case of anatomical variation in the right thigh. The deep femoral vessels and their first perforating vessels pass through the hiatus formed by the pectineus. The pectineal hiatus in the black squares of the schemes **a**, **c** are magnified in the photographs **b**, **d**. The tendon of the superficial layer is indicated by an asterisk (*).*P* pectineus, *AL* adductor longus, *IP* iliopsoas, *Sa* sartorius, *FV* femoral vein, *FA* femoral artery, *DFA* deep femoral artery, *DFV* deep femoral vein, *Per* first perforating vessels, *FN* femoral nerve

During routine dissection of the thigh, bilateral variations in the pectineus muscles were identified in an 82-year-old Korean male cadaver. No signs of previous surgery or injury were observed in this region. On both sides, the superficial and deep layers of the pectineus were divided at its distal part. The superficial part formed its own slim tendon, of which the fibers descended and intermingled with the aponeurosis of the adductor longus distally, and the common aponeurosis inserted into the linea aspera. The tendon of the deep part descended and was attached to the pectineal line. Grossly, a triangular-shaped hiatus was formed by tendons of the two discrete layers and the femur shaft (Fig. 1). On the left side, the deep femoral artery pierced the hiatus, and then, the first perforating artery was branched from the deep femoral artery (Fig. 1a,b). The pectineus muscle was detached from its attachment sites and the lengths of muscular slips were measured with a flexible plastic ruler. The slip lengths of the superficial and deep layers were about 6.2 cm and 3.6 cm, respectively. On the right side, the first perforating artery was branched from the deep femoral artery before approaching the hiatus, and both arteries passed through the hiatus together (Fig. 1c, d). The slip lengths of the superficial and deep layers were about 5 cm and 2.5 cm, respectively. The courses of the deep femoral artery and the first perforating artery below the hiatus were similar to those reported previously. They coursed down between the superficial part of the pectineus and the adductor brevis, and then the adductor longus and the latter. The corresponding veins accompanied the arteries. On both sides, the pectineus was solely innervated by the femoral nerve at its proximal part. The branch to the pectineus arose from the femoral nerve distal to the inguinal ligament and coursed posteriorly to the femoral vessels and their sheath to innervate the pectineus on its anterior surface. No innervation by the branch from the obturator or the accessory obturator nerve was identified.

## Discussion

Various types of double or divided adductor group muscles have been reported. The adductor longus is occasionally double (Standring 2016). The adductor minimus, completely distinct from the adductor magnus, was observed in 52.5% of the thighs by Tubbs et al. In the same study, partially divided muscles were also observed in 24% of the thighs (Tubbs et al. 2011). In addition, the supernumerary muscle, having a probable developmental origin from the obturator externus, was described (Nakamura et al. 1992). However, to the best of our knowledge, variation in the pectineus forming a hiatus by separation of its two insertion sites has not been reported yet.

There have been a few reported variations of the pectineus associated with the adductor longus. Bardeen described the occasional fusion of the pectineus with the adductor longus, but did not identify the exact part and the frequency (Bardeen 1906). Kumar observed pectineus and adductor longus muscle with a common origin from the superior ramus of pubis (Kumar 2013). However, the completely discrete insertion of the pectineus, particularly the insertion of the superficial layer into the tendon of the adductor longus, has not been reported.

In a morphogenetic study with mouse thigh, Jones observed the pectineus muscle with two different anlagen in the embryonic mouse, one of which was from the adductor group and the other was from the iliopsoas. These two anlagen were united in late prenatal development and formed the pectineus. During this period, myoblastic and myotubular condensation as a single unit in the pectineus and the adductor longus was observed (Jones 1979). By reviewing the previous literature on comparative anatomy, Paterson explained that two incompletely separated strata of the human pectineus had different origins. Whereas the superficial stratum of the pectineus was closely associated with the dorsal premuscle mass and innervated by the femoral nerve, the deep stratum was from the ventral mass and occasionally innervated by the obturator nerve (Paterson 1891). This theory was supported by the human embryogenic findings by Gräefenberg and Bardeen. In human embryo on week 6, Gräefenberg identified that the iliopsoas, pectineus, and adductor longus differentiated from a single mass. Pectineus, developing as the border muscle in this mass, was innervated by the femoral nerve laterally and by the obturator nerve medially (Gräefenberg, 1904). Bardeen, via observing leg muscle development in a 14-mm human embryo, identified that the pectineus was separated by a small interval from the iliopsoas and more closely associated with the anlage of the adductor longus (Bardeen 1906). Considering these reports, it is possibly inferred that the human pectineus is also formed by union of the iliopsoas and adductor group anlagen, and then separated from these anlagen during embryogenic development. As a result, two layers with different origins inside the pectineus were formed. Therefore, the hiatus formed by the two layers of the pectineus observed in this study was the first ontogenetic vestige found in adult specimen, which implies the incomplete union of two different anlagen during myogenic development. The insertion of the superficial layer into the adductor longus tendon can be explained by the close association between the pectineus and the adductor longus in prenatal development.

In this case, innervation by the obturator branch to the pectineus was not identified despite careful investigation. A branch from the femoral nerve was the only innervation to both superficial and deep layers of the muscle. This is in accordance with the results of Paterson and Bardeen. They suggested that two strata of the pectineus had different origins, but failed to trace a branch from the obturator nerve innervating the muscle in their human embryo specimens (Bardeen 1906; Paterson 1891). On the contrary, Gräefenberg identified that the pectineus was dual-innervated by the femoral nerve laterally and by the obturator nerve medially in human embryo. To understand this contradiction, we focused on the Gräefenberg’s finding that branches from the femoral and obturator nerves formed a loop between these nerves before the emergence of the pectineus in an 11-mm human embryo. This embryogenic stage precedes the stages investigated by Paterson and Bardeen. By some reasons, this nervous loop, particularly the obturator part, might have regressed and only the branch from the femoral part remained as it supplied the pectineus. Even though this hypothesis is not verified by present embryological knowledge, this possibly explains the obturator innervation to the pectineus identified by Anagnostopoulou et al. with frequency of 4.8% in adult cases (Anagnostopoulou et al. 2009). In these cases, it is inferred that the obturator part of the nervous loop might not have regressed, so remained as variation structures as supplying the pectineus.

Veith et al. suggested an anteromedial vascular approach to access the distal parts of the deep femoral artery when the approach to more proximal vessels is limited (Veith et al. 2016). In this surgical route, the adductor longus should be reflected medially to expose the distal part of the deep femoral artery. However, in a patient with *pectineal hiatus,* where the deep femoral artery passes through the pectineal hiatus, retraction of the muscle medially located to the deep femoral artery would be limited. The pectineal hiatus identified in this case was not spatially enough for surgical approach even on the left side with longer slips. Furthermore, forceful retraction might damage the pectineus in this case. Considering pectineus injury is a cause of acute groin pain (Serner et al. 2018), this muscle should be preserved from intraoperative damage for the quality-of-life of patients. Therefore, understanding the anatomic variations in the pectineus and their positional relationship to the deep femoral artery is important for surgeons.

Anatomical studies on the pectineus are relatively scarce. The pectineus muscle has been regarded as a muscle with few variations and less isolated functional contributions than the other adductor muscle group (Paterson 1891; Standring 2016). However, with the advancement in surgical and anesthetic techniques, the clinical importance of the muscle has been revisited recently (Kubo et al. 2015; Ueshima et al. 2020). Follow-up studies on the pectineus, particularly on its possible variations with frequency and on innervation patterns, are required.
